# *GhLUX1* and *GhELF3* Are Two Components of the Circadian Clock That Regulate Flowering Time of *Gossypium hirsutum*

**DOI:** 10.3389/fpls.2021.691489

**Published:** 2021-08-09

**Authors:** Pengbo Hao, Aimin Wu, Pengyun Chen, Hantao Wang, Liang Ma, Hengling Wei, Shuxun Yu

**Affiliations:** ^1^College of Agronomy, Northwest A&F University, Yangling, China; ^2^State Key Laboratory of Cotton Biology, Institute of Cotton Research of CAAS, Anyang, China

**Keywords:** *GhLUX1*, *GhELF3*, circadian clock, cotton, flowering time

## Abstract

Photoperiod is an important external factor that regulates flowering time, the core mechanism of which lies in the circadian clock-controlled expression of *FLOWERING LOCUS T (FT)* and its upstream regulators. However, the roles of the circadian clock in regulating cotton flowering time are largely unknown. In this study, we cloned two circadian clock genes in cotton, *GhLUX1* and *GhELF3*. The physicochemical and structural properties of their putative proteins could satisfy the prerequisites for the interaction between them, which was proved by yeast two-hybrid (Y2H) and Bimolecular Fluorescent Complimentary (BiFC) assays. Phylogenetic analysis of LUXs and ELF3s indicated that the origin of LUXs was earlier than that of ELF3s, but ELF3s were more divergent and might perform more diverse functions. *GhLUX1, GhELF3, GhCOL1*, and *GhFT* exhibited rhythmic expression and were differentially expressed in the early flowering and late-flowering cotton varieties under different photoperiod conditions. Both overexpression of *GhLUX1* and overexpression of *GhELF3* in *Arabidopsis* delayed flowering probably by changing the oscillation phases and amplitudes of the key genes in the photoperiodic flowering pathway. Both silencing of *GhLUX1* and silencing of *GhELF3* in cotton increased the expression of *GhCOL1* and *GhFT* and resulted in early flowering. In summary, the circadian clock genes were involved in regulating cotton flowering time and could be the candidate targets for breeding early maturing cotton varieties.

## Introduction

Floral transition under favorable circumstances is necessary for the reproductive success of most plant species. Changes in day length (photoperiod) are reliable environmental signals that can be monitored by plants to ensure the proper flowering time (Song et al., 2013; Shim et al., 2017). Generally, the photoperiodic flowering pathway can be divided into three domains: light input, circadian clock, and output. *CONSTANS* (*CO*), the key activator of *FLOWERING LOCUS T* (*FT*), is regulated by both light signaling and the circadian clock. The circadian clock restricts *CO* transcription to late afternoon and night. In long days (LD), CO protein is stabilized by the light of late afternoon and activate the transcription of *FT*, In short days (SD), CO protein is degraded at night and *FT* transcription can’t be activated, which leads to late flowering (Kinmonth-Schultz et al., 2013).

The molecular architecture of the *Arabidopsis* circadian clock is comprised of multiple feedback loops. The initial model is a transcriptional feedback loop comprised of *LATE ELONGATED HYPOCOTYL (LHY)*, *CIRCADIAN CLOCK ASSOCIATED 1 (CCA1)* and *TIMING OF CAB EXPRESSION 1 (TOC1)*. In the morning, *LHY* and *CCA1* are expressed and repressed *TOC1* transcription (Alabadi et al., 2001; Lu et al., 2009; Yakir et al., 2009). At dusk, the decreased levels of *CCA1* and *LHY* induce *TOC1* expression, which in turn represses *CCA1* and *LHY* transcription (Gendron et al., 2012; Huang et al., 2012). An additional loop is comprised of *PSEUDO-RESPONSE REGULATOR 9 (PRR9), PRR7* and *PRR5*, which are sequentially expressed throughout the day and redundantly repressed *CCA1* and *LHY* expression (Nakamichi et al., 2010; Nakamichi et al., 2012; Liu et al., 2016). *PRR9, PRR7* and *PRR5* are reciprocally repressed by *CCA1* and *LHY* (Adams et al., 2015). In addition, *PRR9* is also repressed by the evening complex (EC) (Nagel and Kay, 2012), which is comprised of *LUX ARRHYTHMO (LUX)*, and *EARLY FLOWERING 3 (ELF3)* and *ELF4*. The repression of the EC components by *CCA1, LHY* and the activation of *CCA1, LHY* by the EC components form another feedback loop (Nagel and Kay, 2012; Adams et al., 2015).

Since the circadian clock is comprised of multiple interconnected feedback loops, mutation and overexpression of any component of the circadian clock will change the oscillation properties (phase, period and amplitude) of other components and affect flowering time. In *Arabidopsis*, both *cca1* mutant and *lhy* mutant show early flowering only under SD conditions (Mizoguchi et al., 2002), while *cca1 lhy* double mutant shows early flowering under both LD and SD conditions (Mizoguchi et al., 2002; Fujiwara et al., 2008). Both *CCA1* overexpression and *LHY* overexpression delay flowering under both LD and SD conditions (Wang and Tobin, 1998; Mizoguchi et al., 2002; Lu et al., 2012). Both *prr5* mutant and *prr7* mutant show late flowering only under LD conditions (Yamamoto et al., 2003; Nakamichi et al., 2005, 2007), and *prr5 prr7 prr9* triple mutant also shows late flowering only under LD conditions (Nakamichi et al., 2005, 2007). Both *PRR5* overexpression and *PRR9* overexpression promote flowering under both LD and SD conditions (Matsushika et al., 2002; Sato et al., 2002). Mutation of any EC component (*ELF3, ELF4, LUX*) promotes flowering more significantly under SD conditions than under LD conditions (Zagotta et al., 1996; Liu et al., 2001; Doyle et al., 2002; Hazen et al., 2005; Lu et al., 2012). Both *ELF3* overexpression and *ELF4* overexpression delay flowering under only LD conditions (Liu et al., 2001; McWatters et al., 2007).

The effects of the circadian clock on flowering time have also been reported in some crops. In barley, *PHOTOPERIOD1 (Ppd-H1)* gene, a homolog of *AtPRR7*, regulates photoperiodic flowering by promoting *HvFT1* expression independently of *HvCO1* (Turner et al., 2005; Campoli et al., 2012). Loss-of-function of *HvELF3* leads to early flowering under both LD and SD conditions. *HvELF3* also plays key roles in maintaining the photoperiodic sensitivity in spring barley by repressing *HvFT1* (Faure et al., 2012; Boden et al., 2014). In rice, overexpression of *OsCCA1* leads to late flowering (Izawa et al., 2002; Murakami et al., 2007). Loss-of-function of *OsPRR37*, a homolog of *AtPRR7*, promotes flowering. Overexpression of *OsPRR37* delays flowering (Liu et al., 2018). *OsELF3*, promotes flowering in SDs by activating *OsEhd1* and promotes flowering in LDs by repressing *OsGhd7* (Zhao et al., 2012). In soybean, overexpression of *GmPRR37* delays flowering and mutation of *GmPRR37* promotes flowering under LD conditions (Wang et al., 2020). Overexpression of *GmELF4* in *Arabidopsis* delays flowering (Marcolino-Gomes et al., 2017).

Upland cotton (*Gossypium hirsutum*) is an important cash crop for its high productivity of natural textile fiber, seed oil and protein meal (Zhang T. et al., 2015). With the increasing competition for farmland use between cotton and grain, early maturation of cotton has become a primary breeding objective to enable cotton-wheat rotation. In addition, shortened life cycle allows cotton plants to develop under suitable climatic conditions (Li et al., 2013). However, little is known about the molecular mechanisms that regulate the flowering time of cotton. Recent studies report that the two integrators of multiple flowering pathways, *GhFT* and its putative activator, *GhCOL1 (CONSTANS-like 1)*, are overexpressed in *Arabidopsis* and the transgenic plants exhibit early flowering. Moreover, both *GhCOL1* and *GhFT* exhibit diurnally rhythmic expression with peak in the morning (Guo et al., 2015; Cai et al., 2017). These observations imply that the circadian clock is involved in regulating cotton flowering time. In our study, two circadian clock components, *GhLUX1* and *GhELF3* were cloned. The physicochemical properties and tertiary structures of their protein sequences were predicted. We further analyzed the rhythmic expression patterns of *GhLUX1, GhELF3, GhCOL1*, and *GhFT* in the early flowering and late-flowering varieties under different photoperiod conditions. Finally, we characterized the roles of *GhLUX1* and *GhELF3* in regulating flowering time by overexpressing their coding sequences in *Arabidopsis* and silencing their transcripts in cotton. This work demonstrates that the circadian clock is involved in regulating cotton flowering time for the first time and lays a foundation for exploring how the interaction of multiple flowering pathways controls cotton flowering time.

## Materials and Methods

### Plant Materials and Growth Conditions

The early flowering cotton variety CCRI50 and the late-flowering cotton variety GX11 (Cheng et al., 2021) were grown in the constant temperature (25°C) room under the LD cycles (16 h light/8 h dark). When the fifth true leaves of cotton seedlings were fully expanded, the seedlings of CCRI50 and GX11 were divided into four portions. One portion was remained in the room under the LD cycles and the other three portions were transferred into the rooms under the SD cycles (8h light/16h dark), constant dark and constant light at 6:00, respectively. After the seedlings were entrained for 24 h under the four conditions, the first true leaves of three biological replicates of the seedlings were sampled every 4 h from 6:30 to 2:30 of the next day to extract RNA. Cotton variety GX11 were grown in the constant temperature (25°C) room under the LD cycles (16 h light/8 h dark). The seedlings at the cotyledon stage were used for VIGS experiment. Positive VIGS plants’ first and second true leaves were defoliated when the fourth true leaves were fully expanded. When the eighth true leaves were fully expanded, the fourth true leaves of three biological replicates of positive VIGS plants were sampled every 4 h from 6:30 to 2:30 of the next day to extract RNA.

To produce the plants used for genetic transformation, sterilized *Arabidopsis thaliana* (Columbia ecotype) seeds were sown on the 1/2 MS media with 0.8% agar, and after incubation at 4°C for 3 days, the plates were placed in the constant temperature (21°C) room under the LD cycles (16 h light/8 h dark). Ten-days-old seedlings were transplanted into pots and cultivated in the same room. The T_3_ lines of *GhLUX1*-overexpressed and *GhELF3*-overexpressed *Arabidopsis* and WT were grown under the same conditions to observe their phenotypes of flowering time, bolting time and rosette leave number. When the WT plants’ flower buds were visible, the top fourth rosette leaves of three biological replicates of WT, *GhLUX1*-overexpressed and *GhELF3*-overexpressed *Arabidopsis* seedlings were sampled every 3 h from 7:00 to 4:00 of the next day to extract RNA.

Tobacco (*Nicotiana benthamiana*) was grown in the constant temperature (21°C) room under the LD cycles (16 h light/8 h dark). Five-weeks-old tobacco plants were used for subcellular localization and BiFC experiments.

### Gene Cloning and Sequence Analysis

The protein sequences of AtLUX1 (AT3G46640) and AtELF3 (AT2G25930) were, respectively, used as the queries to search against the protein databases of *G. hirsutum*^1^ using BLAST with *e*-value threshold set at 1e-5. The best hits were defined as GhLUX1 and GhELF3, respectively. The coding sequences of *GhLUX1* and *GhELF3* were amplified from the cDNA of the cotton varieties TM-1, CCRI50 and GX11, and the genomic sequences of *GhLUX1* and *GhELF3* were amplified from the DNA of the cotton variety TM-1 using the gene-specific primers (Supplementary Table 2). The PCR products were cloned into the pBI121 vector and sequenced. The exon-intron structures of *GhLUX1* and *GhELF3* were generated and visualized by submitting their genomic and coding sequences to GSDS 2.0^2^ (Hu et al., 2015). The molecular weight, isoelectric point and grand average of hydropathicity of *GhLUX1*’s and *GhELF3*’s putative protein sequences were predicted using ExPASy^3^ (Artimo et al., 2012).

The protein sequences of AtLUX1 and AtELF3 were, respectively, used as the queries to search against the protein databases of 27 plant species (Supplementary Table 3) using BLAST with *e*-value threshold set at 1e-5. BLAST hits with scores more than 200 were considered as homologs of AtLUX1 and AtELF3. The protein sequences of all the LUXs and ELF3s were, respectively, aligned using Clustal Omega with default parameters^4^ (Madeira et al., 2019). The resulted alignments were used as the input files of MrBayes v3.2.5 to construct the phylogenetic trees with the evolutionary model set to the GTR substitution model and Ngen, Samplefreq set to 1,000,000, 100, respectively (Ronquist and Huelsenbeck, 2003).

The tertiary structures of GhLUX1 and GhELF3 were predicted on the I-TASSER website^5^ (Roy et al., 2010). The multiple sequence alignment results of all the LUXs and ELF3s were, respectively, used to calculate conservation scores of each amino acid site of GhLUX1 and GhELF3 on the Protein Residue Conservation Prediction website^6^ with the default parameters (Capra and Singh, 2007). The tertiary structures were visualized using PyMOL v2.3.0 and the conservation score of each amino acid site was mapped to the color of corresponding amino acid of the tertiary structures with blue corresponding to low conservation score and red corresponding to high conservation score.

### DNA, RNA Extraction, and Quantitative Real-Time PCR (qRT-PCR)

Genomic DNA was extracted via the cetyl-trimethylammonium bromide (CTAB) method as described previously (Porebski et al., 1997). Total RNA was isolated using an RNAprep Pure Plant Kit (DP441) (Tiangen, Beijing, China). The RNA was used as the template for cDNA synthesis using a PrimeScript^TM^ RT Reagent Kit with gDNA Eraser (RR047A) (TaKaRa, Dalian, China). The qRT-PCR was performed using UltraSYBR Mixture (Low ROX) (CW2601) (CWBIO, Beijing, China) and an ABI 7500 Real-Time PCR System (Applied Biosystems, Foster City, CA, United States). The thermocycler program consisted of pre-denaturation at 95°C for 30 s followed by 40 cycles at 95°C for 10 s, 60°C for 30 s, and 72°C for 32 s. The data were calculated in accordance with the 2^–ΔΔCt^ formula, in which ΔΔCt = Ct_gene_ – Ct_reference_ – scale factor (the maximum of Ct_gene_ – Ct_reference_ of all the samples in one experiment) (Livak and Schmittgen, 2001). *GhActin* and *AtACT2* were, respectively, used as the reference genes when analyzing samples of cotton and *Arabidopsis*. The gene-specific primers used for the qRT-PCR were listed in the Supplementary Table 2.

### Transcription Activation and Y2H Assays

The full-length, N-terminal and C-terminal coding sequences of *GhLUX1* and *GhELF3* were cloned into the pGBKT7 and pGADT7 vectors with the gene-specific primers (Supplementary Table 2). Then, the combinations of pGADT7 with pGBKT7, pGBKT7-*GhLUX1*, pGBKT7-*GhELF3*, pGBKT7-*GhLUX1-N*, and pGBKT7-*GhLUX1-C* were co-transferred into the yeast strain Y2HGold which was cultured on DDO (SD/-Leu/-Trp) plates for 3 days. Three independent colonies on the DDO plates were chosen to test the transcription activations on QDO (SD/-Leu/-Trp/-His/-Ade) plates. The combinations of pGADT7, pGADT7-*GhELF3*, pGADT7-*GhELF3-N*, pGADT7-*GhELF3-C* with pGBKT7, pGBKT7-*GhLUX1-C* were co-transferred into the yeast strain Y2HGold which was cultured on DDO plates for 3 days. Three independent colonies on the DDO plates were chosen to detect the interactions on QDO plates.

### Subcellular Localization and BiFC Assays

The coding sequences of *GhLUX1* and *GhELF3* were cloned into the pBI121-*GFP* vectors with the gene-specific primers (Supplementary Table 2). The recombinant vectors were transiently transformed into the leaves of 5-weeks-old tobacco plants using *Agrobacterium tumefaciens* strain GV3101. After the plants were placed in the dark for 2 days, the injected leaves’ fluorescence was observed using confocal laser scanning microscopy (Leica TCS SP8).

The coding sequences of *GhLUX1* and *GhELF3* were, respectively, cloned into the pSPYCE and pSPYNE vectors with the gene-specific primers (Supplementary Table 2). *Agrobacterium* solutions containing pSPYCE, pSPYNE and pSPYCE-*GhLUX1* were mixed with the same volumes of *Agrobacterium* solutions containing pSPYNE-*GhELF3*, pSPYCE-*GhLUX1*, and pSPYNE-*GhELF3*, correspondingly. The following procedures were same to those used in the above subcellular localization experiment.

### *Arabidopsis* Transformation

The recombinant pBI121 vectors (pBI121-*GhLUX1* and pBI121-*GhELF3*) constructed in the gene cloning step were transferred into the *Agrobacterium tumefaciens* strain GV3101 and were transformed into *Arabidopsis* via the floral dip method (Clough and Bent, 1998). The positive plants were selected on 1/2MS medium containing kanamycin (50 mg/L), and further confirmed via PCR and qRT-PCR.

### Virus-Induced Gene Silencing

Virus-induced gene silencing (VIGS) was performed as described previously (Gu et al., 2014). Briefly, the ∼300 bp fragments within *GhLUX1*’s and *GhELF3*’s coding sequences were cloned into the pCLCrVA vector using gene-specific primers (Supplementary Table 2). The recombinant vectors were transferred into the *Agrobacterium tumefaciens* strain GV3101. Solutions of *Agrobacterium* containing pCLCrV-*GhLUX1*, pCLCrV-*GhELF3*, pCLCrV-*PDS* (positive control), pCLCrVA (negative control) were, respectively, mixed with solutions of *Agrobacterium* containing pCLCrVB (helper vector). The mixed solutions were injected into the cotyledons of 10-d-old GX11 seedlings. When the leaves of the pCLCrVA-*PDS* plants became white, positive plants were detected using PCR and qRT-PCR, and then the positive plants were transplanted into large pots and used for phenotypic observation of flowering time.

## Results

### Characterization of Nucleotide and Putative Protein Sequences of GhLUX1 and GhELF3

The most homologous genes in *G. hirsutum* to *AtLUX* and *AtELF3* were identified as *GhLUX1* and *GhELF3*, respectively. The coding sequences of *GhLUX1* and *GhELF3* cloned from CCRI50 and GX11 were same to those cloned from TM-1, suggesting that the protein functions of GhLUX1 and GhELF3 might be unchanged in different cotton varieties. By comparing the coding sequences and genomic sequences, one exon and four exons were found in *GhLUX1* and *GhELF3*, respectively (Supplementary Figure 1). The properties of putative protein sequences were listed in Supplementary Table 1. Notably, the isoelectric points (pIs) of GhLUX1 and GhELF3 were 5.28 and 8.84, respectively, suggesting they were charged oppositely in cotton cells. In addition, GhLUX1 and GhELF3 showed similar grand average of hydropathicity (GRAVY) and were both hydrophilic proteins. These properties of GhLUX1 and GhELF3 satisfied some prerequisites for the interaction between the two proteins.

### Evolutionary Difference Between LUXs and ELF3s

To explore the evolutionary difference between LUXs and ELF3s, homologs of AtLUX and AtELF3 were screened in 27 plant species’ protein databases and the phylogenetic trees were constructed. There was no LUX identified in chlorophytes (*C. reinhardtii*) and bryophytes (*P. patens*). The most ancient LUX was identified in pteridophytes (*S. moellendorffii*). Only one LUX was found in the early species before dicots, while one to six LUXs were found in different dicots. More than one LUXs contained in some dicots (*G. max, P. trichocarpa, D. carot, A. thaliana, B. rapa* and four *Gossypium* species) had the closest phylogenetic relationships (Supplementary Figure 2A). The most ancient ELF3 was identified in the most basal lineage of angiosperms (*A. trichopoda*). The numbers of ELF3s increased to two or three in monocots and ELF3s in dicots diverged into two subclades (Supplementary Figure 2B). These results indicated that ELF3s might arise later than LUXs, but evolve more rapidly to perform more diverse functions in plants than LUXs.

### Characterization of the Predicted Tertiary Structures of GhLUX1 and GhELF3

The tertiary structure of one protein usually implies its potential molecular functions. The tertiary structures of GhLUX1 and GhELF3 were predicted on the I-TASSER server and their conservation scores at each amino acid site were calculated on the Protein Residue Conservation Prediction website. GhLUX1 consisted of helices and coils. Two conserved regions were distributed in the N-terminus and middle part of the protein, respectively. The more conserved Myb DNA-binding domain consisted of three helices (Supplementary Figure 3A). GhELF3 was divided into an N-terminal large subunit and a C-terminal small subunit linked by a random coil. There was large open space between the large subunit and the small subunit. The helices and coils of the large subunit formed a groove, the two terminals of which were two conserved regions. The small unit consisted of helices, sheets and coils and contained two close conserved regions in its middle part (Supplementary Figure 3B).

### Transcriptional Activity and Interaction of GhLUX1 and GhELF3

**FIGURE 1:**
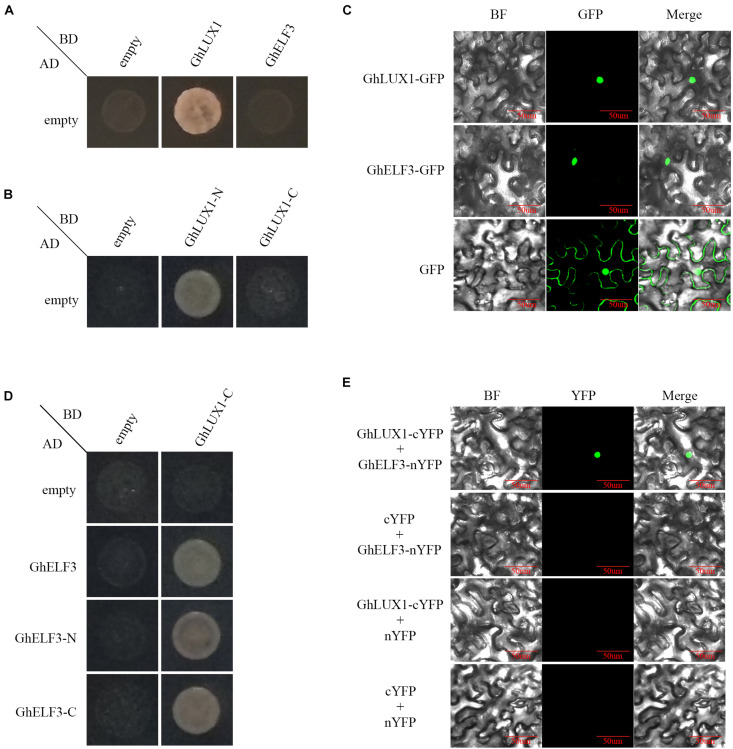
Transcriptional activity and interaction of GhLUX1 and GhELF3. **(A)** Transcriptional activity of GhLUX1 and GhELF3 in Y2HGold yeast cells. Yeast cells containing different combinations of pGADT7 and pGBKT7, pGBKT7-*GhLUX1*, pGBKT7-*GhELF3* vectors are cultured on the SD-Trp/-Leu/-His/-Ade/medium. **(B)** Transcriptional activity of GhLUX1-N and GhLUX1-C in Y2HGold yeast cells. Yeast cells containing different combinations of pGADT7 and pGBKT7, pGBKT7-*GhLUX1-N* (residues 1–154), pGBKT7-*GhLUX1-C* (residues 155–337) vectors are cultured on the SD-Trp/-Leu/-His/-Ade/medium. **(C)** Subcellular localization of GhLUX1 and GhELF3 in *N. benthamiana* epidermal cells. GhLUX1-GFP, GhELF3-GFP, and GFP are transiently expressed in *N. benthamiana* epidermal cells and visualized under confocal microscopy. **(D)** GhLUX1-GhELF3 interaction in Y2HGold yeast cells. Yeast cells containing different combinations of pGADT7, pGADT7-*GhELF3*, pGADT7-*GhELF3-N* (residues 1–460), pGADT7-*GhELF3-C* (residues 467–705) and pGBKT7, pGBKT7-*GhLUX1-C* vectors are cultured on the SD-Trp/-Leu/-His/-Ade/medium. **(E)** GhLUX1-GhELF3 interaction in *N. benthamiana* epidermal cells. Different combinations of *GhLUX1*-cYFP, *GhELF3*-nYFP and empty vectors are transiently coexpressed in *N. benthamiana* epidermal cells and visualized under confocal microscopy.

To examine whether GhLUX1 and GhELF3 acted as transcription factors, the transcriptional activation assay was performed in yeast cells. The yeast cells containing pGADT7 and pGBKT7-GhLUX1 plasmids could grow normally on the quadruple dropout media, whereas the yeast cells containing pGADT7 and pGBKT7-GhELF3 plasmids could not (Figure 1A), suggesting that GhLUX1 had transcriptional activity, but GhELF3 did not. Further segmentation of GhLUX1 suggested GhLUX1-N (residues 1–154) had transcriptional activity, but GhLUX1-C (residues 155–337) did not (Figure 1B). Subcellular localization assay showed that both GhLUX1-GFP and GhELF3-GFP could be transported into the nucleus of *N. benthamiana* cells (Figure 1C), indicating that both GhLUX1 and GhELF3 might perform their functions in the nucleus.

In *Arabidopsis*, the evening complex (EC) was formed by the direct interactions of AtELF3 and AtLUX (residues 144–323), AtELF3 (residues 261–484) and AtELF4 (Huang and Nusinow, 2016). To examine whether GhLUX1 interacted with GhELF3, yeast two-hybrid and Bimolecular Fluorescent Complimentary (BiFC) assay were performed. Because of the auto-activations of GhLUX1 and GhLUX1-N (Figures 1A,B), GhLUX1-C was used as the bait. GhLUX1-C showed interactions with GhELF3, GhELF3-N (residues 1–460) and GhELF3-C (residues 467–705) (Figure 1D). The BiFC result showed that GhLUX1 interacted with GhELF3 in the nuclei of *N. benthamiana* cells (Figure 1E). The transcriptional activity of GhLUX1 and the interaction of GhLUX1 and GhELF3 in the nucleus indicated that GhLUX1 might recruit GhELF3 to the promoters of target genes to regulate their transcriptions.

### Rhythmic Expression of *GhLUX1, GhELF3, GhCOL1*, and *GhFT* in LD and SD

**FIGURE 2:**
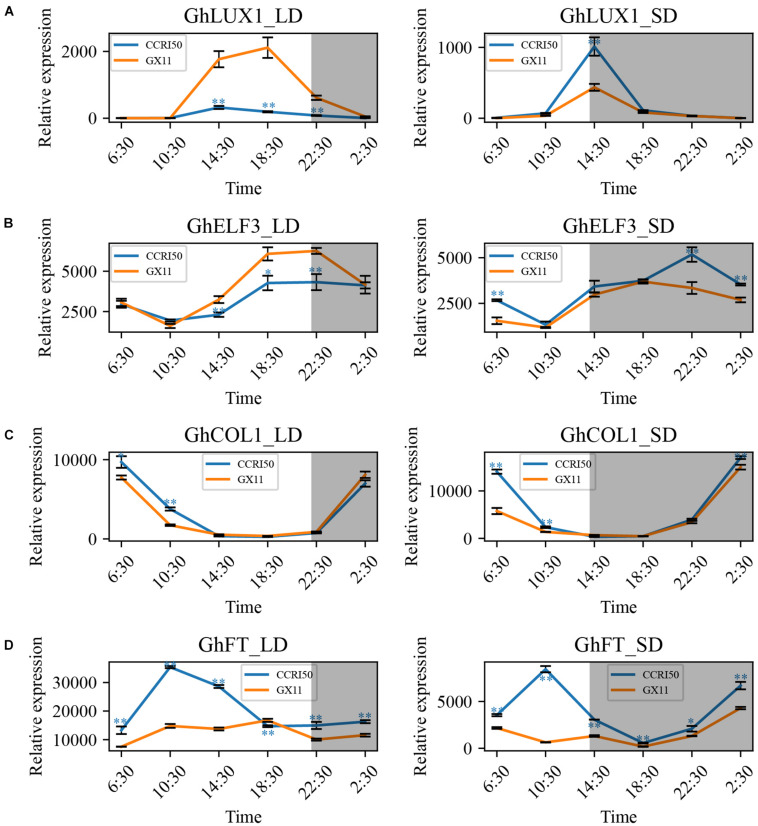
*GhLUX1, GhELF3, GhCOL1*, and *GhFT* are differentially expressed in late-flowering GX11 and early flowering CCRI50 under LD and SD conditions. Expression patterns of **(A)** *GhLUX1*, **(B)** *GhELF3*, **(C)** *GhCOL1*, and **(D)** *GhFT* in GX11, CCRI50 under LD and SD conditions. All the expression levels are made relative to the expression level of *GhLUX1* in GX at 2:30 under SD. The data are the means ± standard errors (SEs) of three biological replicates. The asterisks indicate significant differences of comparison between GX11 and CCRI50 at each time point (^∗∗^*P* < 0.01, ^∗^*P* < 0.05, Student’s *t*-test). The gray shadows indicate the dark periods.

To determine whether cotton flowering time was regulated by *GhLUX1* and *GhELF3*, the expression patterns of *GhLUX1, GhELF3, GhCOL1*, and *GhFT* in LD (16 h light/8 h dark) and SD (8 h light/16 h dark) were compared between the early flowering variety, CCRI50 and the late-flowering variety, GX11. All the four genes exhibited rhythmic expression patterns under both photoperiod conditions and in both cotton varieties (Figure 2). Compared with GX11, CCRI50 showed lower expression levels of *GhLUX1* and *GhELF3* from the afternoon till the early night of LD but showed higher expression levels of *GhLUX1* in the afternoon of SD and higher expression levels of *GhELF3* from the night till the morning of SD (Figures 2A,B), which suggested that *GhLUX1* and *GhELF3* might repress flowering in LD but promote flowering in SD. This situation was similar to that *LUXs* and *ELF3s* repressed flowering in long day plant (LDP) species but promoted flowering in short day plant (SDP) species (Bu et al., 2021). In addition, CCRI50 showed higher expression levels of *GhCOL1* in the morning of both LD and SD and higher expression levels of *GhFT* at most times of both LD and SD (Figures 2C,D), which was consistent with the roles of *GhCOL1* and *GhFT* in promoting flowering.

### Rhythmic Expression of *GhLUX1, GhELF3, GhCOL1*, and *GhFT* in Constant Light and Dark

**FIGURE 3:**
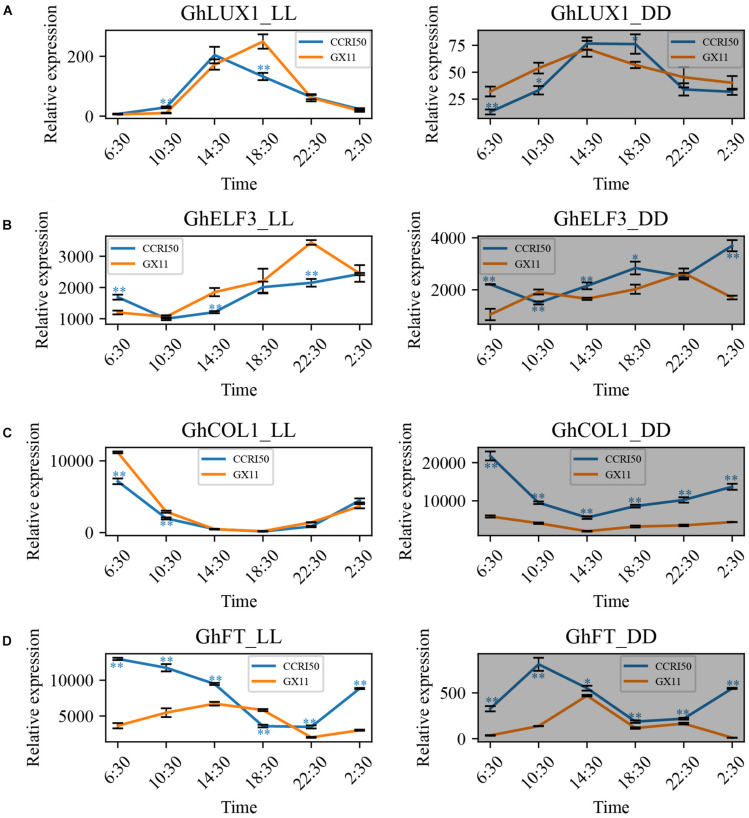
*GhLUX1, GhELF3, GhCOL1*, and *GhFT* persist differently rhythmic expression in late-flowering GX11 and early flowering CCRI50 under constant conditions. Expression patterns of **(A)** *GhLUX1*, **(B)** *GhELF3*, **(C)** *GhCOL1*, and **(D)** *GhFT* in GX11, CCRI50 under LL and DD conditions. All the expression levels are made relative to the expression level of *GhLUX1* in GX at 2:30 under SD. The data are the means ± SEs of three biological replicates. The asterisks indicate significant differences of comparison between GX11 and CCRI50 at each time point (^∗∗^*P* < 0.01, ^∗^*P* < 0.05, Student’s *t*-test). The gray shadows indicate the dark periods.

To exclude the effects of day-night alteration on the oscillations of *GhLUX1*, *GhELF3*, *GhCOL1*, and *GhFT* transcripts, the expression patterns of the four genes in constant light (LL) and dark (DD) were analyzed. In LL and DD, all the four genes still exhibited rhythmic expression patterns in GX11 and CCRI50 (Figure 3). Similar to the situations in LD and SD, CCRI50 showed lower expression levels of *GhLUX1* and *GhELF3* at the specific times of LL but showed higher expression levels of *GhLUX1* and *GhELF3* at the specific times of DD (Figures 3A,B). In addition, CCRI50 showed higher expression levels of *GhFT* at most times of LL and DD (Figure 3D). However, CCRI50 showed lower expression levels of *GhCOL1* in the morning of LL but showed much higher expression levels of *GhCOL1* at all the times of DD (Figure 3C). In addition, compared with the expression of *GhLUX1* and *GhELF3* in LD and SD, the expression of *GhLUX1* in LL and DD was impaired dramatically (Figures 2A, 3A), while the expression of *GhELF3* in LL and DD was just changed slightly (Figures 2B, 3B), indicating the robust oscillation of *GhELF3* under different photoperiod conditions. Furthermore, the expression levels of *GhFT* in DD were dramatically decreased compared with those in LD, SD and LL (Figures 2D, 3D), indicating that *GhFT* was repressed by unknown regulators in darkness. In addition, oscillation phases of *GhFT* transcript were significantly different not only between two varieties but also among the four photoperiod conditions (Figures 2D, 3D). These results implied that the circadian clock could exhibit different oscillation properties in different cotton varieties and could be entrained by different photoperiods.

### Both Overexpression of *GhLUX1* and Overexpression of *GhELF3* in *Arabidopsis* Delay Flowering

**FIGURE 4:**
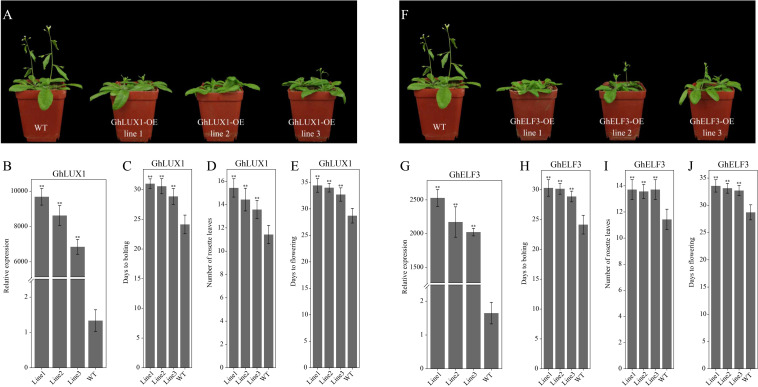
Both overexpression of *GhLUX1* and overexpression of *GhELF3* in *Arabidopsis* delay flowering. **(A,F)** Phenotypic characteristics of 4-week-old WT and transgenic lines. **(B,G)** Expression levels of *GhLUX1* and *GhELF3* in the WT and the transgenic lines. All the expression levels are made relative to the expression level of *GhLUX1* in the WT. The data are the means ± SEs of three biological replicates. **(C,H)** Days to bolting, **(D,I)** rosette leaf numbers and **(E,J)** days to flowering of the WT and the transgenic lines (means ± SEs, *n* = 24 plants). The asterisks indicate significant differences compared to the WT plants at each time point (^∗∗^*P* < 0.01, Student’s *t*-test).

To explore the functional roles of *GhLUX1* and *GhELF3* in regulating flowering time, coding sequences of *GhLUX1* and *GhELF3* driven by the 35S promoter were transformed into *Arabidopsis*. Three independent T_3_ lines with significantly higher expression levels of *GhLUX1* and *GhELF3* than the WT were selected to observe their flowering phenotypes (Figures 4B, 4G). All the transgenic lines exhibited later flowering than the WT did (Figures 4A,F). Compared with the WT, the *GhLUX1*-overexpressed lines and the *GhELF3*-overexpressed lines flowered 4–5.7 and 4–4.9 days later on average, respectively (Figures 4E,J). In addition, the *GhLUX1*-overexpressed lines and the *GhELF3*-overexpressed lines bolted later and had more rosette leaves compared with the WT (Figures 4C,D,H,I), which was consistent with their later flowering time. These results suggested that *GhLUX1* and *GhELF3* could perform similar functions to *AtLUX* and *AtELF3*, respectively, in regulating flowering time of *Arabidopsis* (Zagotta et al., 1996; Hazen et al., 2005).

### Both Overexpression of *GhLUX1* and Overexpression of *GhELF3* Change the Oscillations of the Circadian Clock Genes and the Key Genes in the Photoperiodic Flowering Pathway

**FIGURE 5:**
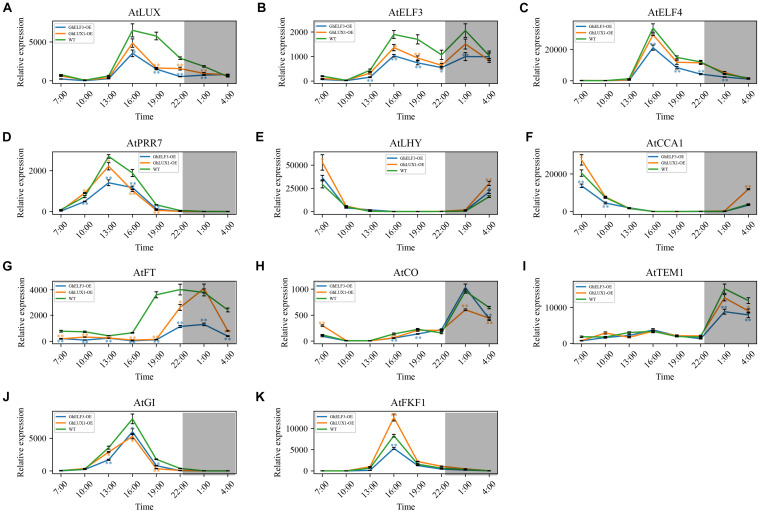
Both overexpression of *GhLUX1* and overexpression of *GhELF3* change the oscillations of the circadian clock genes and the key genes in the photoperiodic flowering pathway. Expression patterns of **(A–F)** the circadian clock genes and **(G–K)** the key genes in the photoperiodic flowering pathway in the WT and the transgenic lines. All the expression levels are made relative to the expression level of *AtFKF1* in the WT at 10:00. The data are the means ± SEs of three biological replicates. The asterisks indicate significant differences compared to the WT plants at each time point (^∗∗^*P* < 0.01, ^∗^*P* < 0.05, Student’s *t*-test). The gray shadows indicate the dark periods.

Because the circadian clock is comprised of multiple interconnected feedback loops, we hypothesized that overexpression of *GhLUX1* and overexpression of *GhELF3* in *Arabidopsis* changed the running of the whole circadian clock. To test the hypothesis, we measured the expression levels of several core circadian clock genes (including *AtLUX, AtELF3, AtELF4, AtPRR7, AtLHY*, and *AtCCA1*) during the 24 h in the transgenic lines and the WT. All the six genes were upregulated or downregulated in the *GhLUX1*-overexpressed line and the *GhELF3*-overexpressed line compared with in the WT, although their expression trends during the 24 h were similar between the two transgenic lines and the WT (Figures 5A–F). Overexpression of *GhELF3* significantly repressed the expression of four evening- or afternoon-phased clock genes (including *AtLUX, AtELF3, AtELF4*, and *AtPRR7*), while overexpression of *GhLUX1* repressed the expression of the four genes to a lesser extent (Figures 5A–D). The expression of a morning-phased gene, *AtLHY*, was promoted in the *GhLUX1-*overexpressed line to a higher extent than in the *GhELF3-*overexpressed line (Figure 5E). The expression of *AtLHY*’s close homolog, *AtCCA1*, was promoted by overexpression of *GhLUX1* but was repressed by overexpression of *GhELF3* (Figure 5F). These results indicated that overexpression of *GhLUX1* and overexpression of *GhELF3* could change the running of the *Arabidopsis* circadian clock differently.

In the important photoperiodic flowering pathway, the key integrators, *CO* and *FT*, as well as a number of their regulators were under the control of the circadian clock. In the *GhLUX1*-overexpressed line and the *GhELF3*-overexpressed line, the expression of *AtFT* was repressed and delayed to the later time of the day (19:00–4:00) compared with in the WT (16:00–4:00). In addition, the expression of *AtFT* was repressed more strongly by *GhELF3* than by *GhLUX1* (Figure 5G). *AtCO*, the primary activator of *AtFT*, exhibited slightly higher expression levels in the WT than in the two overexpression lines at 16:00, which was consistent with the rapidly increasing expression of *AtFT* in WT and the persistent low expression of *AtFT* in the two overexpression lines from 16:00 to 19:00. In addition, *AtCO* exhibited slightly higher expression levels in the WT and the *GhLUX1*-overexpressed line than in the *GhELF3*-overexpressed line at 19:00, which was consistent with the slowly increasing expression of *AtFT* at high level in the WT, the dramatically increasing expression of *AtFT* at medium level in the *GhLUX1*-overexpressed line and the slowly increasing expression of *AtFT* at low level in the *GhELF3*-overexpressed line from 19:00 to 22:00. Although the expression of *AtCO* reached peaks in all the three lines and were repressed in the *GhLUX1*-overexpressed line at 1:00, the expression of *AtFT* began to decrease dramatically in all the three lines and was not repressed in the *GhLUX1*-overexpressed line at 1:00 (Figures 5G,H). This discrepancy between the expression of *AtCO* and *AtFT* at 1:00 might be explained by the degradation of AtCO protein and high expression levels of *AtTEM1* (the main repressor of *AtFT*) at night. The expression of *AtTEM1* began to increase at 22:00 and reached peaks at 1:00. Compared with in the WT, the expression of *AtTEM1* was repressed to a higher extent in the *GhELF3*-overexpressed line than in the *GhLUX1*-overexpressed line (Figure 5I). We speculated that the higher *AtFT* was expressed in the late afternoon and early evening, the higher level of *AtTEM1* was needed to repress the expression of *AtFT* at night. *AtGI* and *AtFKF1* were under the control of the circadian clock, they promoted flowering not only by regulating the expression timing of *AtCO* but also by directly regulating the expression of *AtFT* (Sawa and Kay, 2011). We therefore examined whether the expression of *AtGI* and *AtFKF1* was changed in the *GhLUX1*-overexpressed line and the *GhELF3*-overexpressed line. Compared with in the WT, the expression of *AtGI* was repressed in the *GhLUX1*-overexpressed line and the *GhELF3*-overexpressed line (Figure 5J). The expression of *AtFKF1* was promoted in the *GhLUX1*-overexpressed line but repressed in the *GhELF3*-overexpressed line (Figure 5K). These results suggested that the circadian clock could regulate the diurnally rhythmic expression of the key genes in the photoperiodic flowering pathway to regulate flowering time.

### Both Silencing of *GhLUX1* and Silencing of *GhELF3* in Cotton Promote Flowering

**FIGURE 6:**
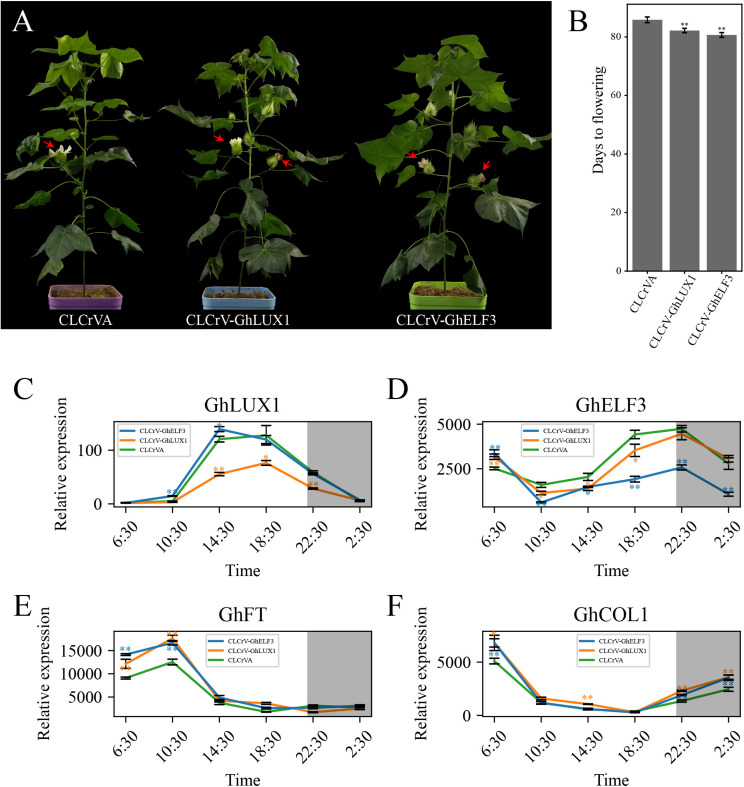
Both silencing of *GhLUX1* and silencing of *GhELF3* in cotton promote flowering. **(A)** Phenotypic characteristics of 86-day-old control (CLCrVA), *GhLUX1*-silenced (CLCrV-*GhLUX1*) and *GhELF3*-silenced (CLCrV-*GhELF3*) plants. **(B)** Days to flowering of the control plants and the gene-silenced plants (means ± SEs, *n* = 18 plants). **(C–F)** Expression patterns of *GhLUX1, GhELF3, GhFT*, and *GhCOL1* in the control plants and the gene-silenced plants. All the expression levels are made relative to the expression level of *GhLUX1* in CLCrV-*GhLUX1* at 6:30. The data are the means ± SEs of three biological replicates. The asterisks indicate significant differences compared to the control plants at each time point (^∗∗^*P* < 0.01, ^∗^*P* < 0.05, Student’s *t*-test). The gray shadows indicate the dark periods.

To further investigate the roles of *GhLUX1* and *GhELF3* in regulating flowering time of cotton, *GhLUX1* and *GhELF3* were silenced in cotton via virus-induced gene silencing (VIGS). The *GhLUX1*-silenced plants and the *GhELF3*-silenced plants flowered 3.6 and 5.1 days earlier on average than the control (CLCrVA) plants (Figure 6B). When the first flowers of the control plants were blooming, the second flowers of the *GhLUX1*-silenced plants and the *GhELF3*-silenced plants were blooming and had bloomed, respectively (Figure 6A). Compared with the control plants, the expression of *GhLUX1* in the *GhLUX1*-silenced plants and the expression of *GhELF3* in the *GhELF3*-silenced plants were significantly decreased when they were highly expressed during the 24 h (Figures 6C,D). In addition, the expression of *GhLUX1* in the *GhELF3*-silenced plants didn’t change, while the expression of *GhELF3* in the *GhLUX1*-silenced plants was slightly repressed at 14:30 and 18:30 (Figures 6C,D). The expression of *GhFT* in both the *GhLUX1*-silenced plants and the *GhELF3*-silenced plants was increased at 6:30 and 10:30, which might result from the increased expression of *GhCOL1* at 2:30 and 6:30 in these plants (Figures 6E,F). These results suggested that the circadian clock might regulate cotton flowering time by regulating the expression of *GhFT* and *GhCOL1*.

## Discussion

Appropriate flowering time is crucial for reproduction success and crop yield. Great efforts have been made to illuminate the complex molecular networks that control flowering time. In *Arabidopsis*, the important photoperiodic flowering pathway depends on the circadian clock-controlled transcription of key genes in the pathway. Here, we report that two components of the circadian clock in cotton, *GhLUX1* and *GhELF3* participate in flowering time regulation by affecting the transcription of *GhCOL1* and *GhFT* (Figure 6).

Circadian clock genes have been found in organisms across the three domains of life: Archaea, Bacteria, and Eucarya. During evolution, reconfiguration of the circadian clock network has led to non-homologous network components utilized by different lineages. The components of transcriptional feedback loops of the clock in early plant lineages (chlorophytes and bryophytes) vs. angiosperms are apparently different (McClung, 2013). Our phylogenetic analysis of LUXs and ELF3s in 27 plant species show that both LUXs and ELF3s are not found in chlorophytes (*C. reinhardtii*) and bryophytes (*P. patens*). The most ancient LUX and ELF3 were identified in pteridophytes (*S. moellendorffii*) and the most basal angiosperm lineage (*A. trichopoda*), respectively (Supplementary Figure 2). These results indicate that LUXs and ELF3s may be sequentially added into the ancestral network of the circadian clock after the occurrence of bryophytes, which is consistent with the hypothesis that the circadian clock network is evolutionarily dynamic with new components joining and old components quitting (McClung, 2013). Given that ELF3 may act as a regulator of light input into the oscillator (McWatters et al., 2000), the occurrence of this novel regulator in the earliest angiosperm implies that light entrainment to the circadian clock may originate from or be reinforced in the higher plant species. Redundancy generated by gene duplication usually promotes regulatory neofunctionalization (redeployment of TFs into new networks) (Wohlbach et al., 2009). ELF3 numbers (two or three) in monocots are more than LUX numbers (only one) in monocots and ELF3s in dicots are divided into two subclades (Supplementary Figure 2). These results suggest that ELF3s are more redundant than LUXs and have diverged more greatly to perform more diverse functions. In *Arabidopsis*, ELF3 doesn’t contain any known DNA-binding domains and therefore performs its regulatory functions mainly by interacting with multiple other proteins, including phyB, COP1, BBX19, PIF4, LUX1, ELF4, NOX, SVP, TOC1, and GI (Huang and Nusinow, 2016). Although we demonstrate that GhELF3 interacts with GhLUX1 in the nucleus (Figures 1D,E), whether GhELF3 can interact with other proteins needs to be further investigated to better understand the diverse roles of GhELF3 in cotton growth and development.

The wild species of cotton are short-day plants that originated from tropical regions (Li et al., 2015). Domesticated *G. hirsutum* became photoperiod-insensitive during its adaptation to long-day conditions of temperate regions, while semi-domesticated races of *G. hirsutum* still are photoperiod-sensitive and don’t flower in LD (Zhang R. et al., 2015). Another short-day plant, *G. max* originated from temperate region. During adaptation to wide latitudes, the photoperiod response of *G. max* is changed due to artificial selection and natural variation of the circadian clock genes (Lu et al., 2017, 2020). We test whether this mechanism leads to different flowering times of cotton cultivars. Both *GhLUX1* and *GhELF3* are differently expressed between CCRI50 and GX11 in LD, SD, LL, and DD (Figures 2A,B, 3A,B), implying that differences in the circadian clock may contribute to different flowering times of cotton cultivars. In addition, the oscillations of *GhLUX1* and *GhELF3* transcripts in CCRI50 and GX11 respond to photoperiod in different manners (Figures 2A,B, 3A,B), indicating that photoperiod may regulate *GhLUX1* and *GhELF3* expression through different ways and photoperiod responses can be different in cotton varieties with different flowering time.

As the integrator of multiple flowering pathways, FT is transported from companion cells of leaves to shoot apical meristem and then induces the expression of floral identity genes (Guo et al., 2015). A previous study demonstrates that *GhFT* also functions as a flowering promoter. The diurnal oscillation of *GhFT* mRNA in both LD and SD implies that the transcription of *GhFT* is under the control of the circadian clock or/and respond to the day-night transition (Guo et al., 2015). Our expression analysis shows that *GhFT* mRNA oscillates diurnally not only in LD and SD, but also in LL and DD (Figures 2D, 3D), which indicates that the circadian clock persists to oscillate and controls the transcription of *GhFT* under constant conditions. The different oscillation properties (the timings of rise and fall during the 24 h, amplitudes, peak levels and trough levels) of *GhFT* mRNA under the four conditions may result from the circadian clock’s response to different photoperiods. However, the oscillation properties of *GhCOL1* mRNA, especially the timing of rise and fall, are similar among the four conditions (Figures 2D, 3D), indicating that *GhCOL1* may be regulated by circadian clock genes different from those regulating *GhFT*. Furthermore, the discrepancy between higher levels of *GhCOL1* and lower levels of *GhFT* in DD suggests that unknown repressors of *GhFT*, probably homologs of *AtTEM1/2* (Castillejo and Pelaz, 2008; Osnato et al., 2012; Marin-Gonzalez et al., 2015), may dominate *GhFT* transcription in the dark. It will be interesting to identify these repressors in cotton and explore whether they are regulated by the circadian clock. In *Arabidopsis*, *AtFT* promoter is directly bound by another circadian clock gene, *AtGI*, and is activated by *GI* in a CO-independent manner (Sawa and Kay, 2011). Further identification of other circadian clock genes in cotton will be helpful to understand the complex roles of the circadian clock in regulating cotton flowering time.

Because the core components of the plant circadian clock form multiple feedback loops and these loops interlocked with one another (Hsu and Harmer, 2014), it’s difficult to confirm the precise molecular functions of one certain component in regulating flowering time. Both overexpression of *GhLUX1* and overexpression of *GhELF3* in *Arabidopsis* alter the oscillation amplitudes of their *Arabidopsis* orthologs and other circadian clock components (Figures 5A–F). Furthermore, the oscillation amplitudes of the core flowering genes in the photoperiodic flowering pathway are also altered in the two transgenic lines, except that the oscillation phase of *AtFT* in the *GhLUX1*-overexpressed line is delayed rather than that the oscillation amplitude is changed (Figures 5G–K). Although GhLUX1 and GhELF3 can perform functions by forming a complex (Figures 1D,E), the different expression alterations of the circadian clock genes and the flowering genes between the *GhLUX1*-overexpressed line and the *GhELF3*-overexpressed line indicate that GhLUX1 and GhELF3 can also perform functions independently from each other. These results are helpful to understand the specific functions of different circadian clock components in orchestrating the expression of multiple flowering genes. Virus-induced silencing of *GhLUX1* and silencing of *GhELF3* in cotton promote flowering by upregulating *GhCOL1* and *GhFT* (Figure 6). Untangling the complex regulation relationships between the circadian clock and flowering in cotton depends on the future identification of direct regulators of *GhCOL1* and *GhFT* in the photoperiodic flowering pathway and other flowering pathways, and more importantly, subsequent investigation of the relationships between the circadian clock and these regulators.

In summary, *GhLUX1* and *GhELF3*, the two components of the circadian clock, are differentially expressed in the early flowering and late-flowering cotton varieties, which also exhibit different expression oscillations of two core flowering genes, *GhCOL1* and *GhFT*. Both overexpression of *GhLUX1* and overexpression of *GhELF3* in *Arabidopsis* delay flowering by altering the expression oscillations of multiple key genes in the photoperiodic flowering pathway. Both silencing of *GhLUX1* and silencing of *GhELF3* in cotton promote flowering by increasing the expression of *GhCOL1* and *GhFT*. Our results demonstrate that the circadian clock is involved in regulating cotton flowering time and provide a theoretical basis for breeding cotton varieties with desired flowering and maturity time.

## Data Availability Statement

The original contributions presented in the study are included in the article/Supplementary Material, further inquiries can be directed to the corresponding author/s.

## Author Contributions

SY and HWe designed the experiments. AW, PC, and LM performed the experiments. PH analyzed the results and wrote the manuscript. HWe and HWa revised the manuscript. All authors reviewed and approved the final manuscript.

## Conflict of Interest

The authors declare that the research was conducted in the absence of any commercial or financial relationships that could be construed as a potential conflict of interest.

## Publisher’s Note

All claims expressed in this article are solely those of the authors and do not necessarily represent those of their affiliated organizations, or those of the publisher, the editors and the reviewers. Any product that may be evaluated in this article, or claim that may be made by its manufacturer, is not guaranteed or endorsed by the publisher.

## Footnotes

1
https://www.cottongen.org/cottongen_downloads/Gossypium_hirsutum/ZJU_G.hirsutum_AD1genome_v2.1/genes/TM-1_V2.1.gene.pep.fa.gz

2
http://gsds.cbi.pku.edu.cn/

3
http://web.expasy.org/protparam/

4
https://www.ebi.ac.uk/Tools/msa/clustalo/

5
https://zhanglab.ccmb.med.umich.edu/I-TASSER/

6
https://compbio.cs.princeton.edu/conservation/score.html

## Supplementary Material

Supplementary Figure 1 Exon-intron structures of *GhLUX1* and *GhELF3*.

Supplementary Figure 2 Phylogenetic trees of LUXs and ELF3s in plant species.

Supplementary Figure 3 Tertiary structures of GhLUX1 and GhELF3.

Supplementary Table 1 Properties of the putative protein sequences of *GhLUX1* and *GhELF3*.

Supplementary Table 2 Primers used in this study.

Supplementary Table 3 Information about the 27 plant species used in identifying LUXs and ELF3s.

## References

[B1] Adams S. Manfield I. Stockley P. Carre I. A. (2015). Revised Morning Loops of the Arabidopsis Circadian Clock Based on Analyses of Direct Regulatory Interactions. *PLoS One* 10:e0143943. 10.1371/journal.pone.0143943 26625126 PMC4666590

[B2] Alabadi D. Oyama T. Yanovsky M. J. Harmon F. G. Mas P. Kay S. A. (2001). Reciprocal regulation between TOC1 and LHY/CCA1 within the Arabidopsis circadian clock. *Science* 293 880–883. 10.1126/science.1061320 11486091

[B3] Artimo P. Jonnalagedda M. Arnold K. Baratin D. Csardi G. De Castro E. (2012). ExPASy: SIB bioinformatics resource portal. *Nucl. Res.* 40 W597–W603. 10.1093/nar/gks400 22661580 PMC3394269

[B4] Boden S. A. Weiss D. Ross J. J. Davies N. W. Trevaskis B. Chandler P. M. (2014). EARLY FLOWERING3 Regulates Flowering in Spring Barley by Mediating Gibberellin Production and FLOWERING LOCUS T Expression. *Plant Cell* 26 1557–1569. 10.1105/tpc.114.123794 24781117 PMC4036571

[B5] Bu T. T. Lu S. J. Wang K. Dong L. D. Li S. L. Xie Q. G. (2021). A critical role of the soybean evening complex in the control of photoperiod sensitivity and adaptation. *Proc. Natl. Acad. Sci. U.S.A.* 8:118. 10.1073/pnas.2010241118 33558416 PMC7923351

[B6] Cai D. Liu H. Sang N. Huang X. (2017). Identification and characterization of CONSTANS-like (COL) gene family in upland cotton (Gossypium hirsutum L.). *PLoS One* 12:e0179038. 10.1371/journal.pone.0179038 28591177 PMC5462432

[B7] Campoli C. Shtaya M. Davis S. J. Von Korff M. (2012). Expression conservation within the circadian clock of a monocot: natural variation at barley Ppd-H1 affects circadian expression of flowering time genes, but not clock orthologs. *BMC Plant Biol.* 12:97. 10.1186/1471-2229-12-97 22720803 PMC3478166

[B8] Capra J. A. Singh M. (2007). Predicting functionally important residues from sequence conservation. *Bioinformatics* 23 1875–1882. 10.1093/bioinformatics/btm270 17519246

[B9] Castillejo C. Pelaz S. (2008). The balance between CONSTANS and TEMPRANILLO activities determines FT expression to trigger flowering. *Curr. Biol.* 18 1338–1343. 10.1016/j.cub.2008.07.075 18718758

[B10] Cheng S. Chen P. Su Z. Ma L. Hao P. Zhang J. (2021). High-resolution temporal dynamic transcriptome landscape reveals a GhCAL-mediated flowering regulatory pathway in cotton (Gossypium hirsutumL.). *Plant Biotechnol. J.* 19 153–166. 10.1111/pbi.13449 32654381 PMC7769237

[B11] Clough S. J. Bent A. F. (1998). Floral dip: a simplified method for Agrobacterium-mediated transformation of Arabidopsis thaliana. *Plant J.* 16 735–743. 10.1046/j.1365-313x.1998.00343.x 10069079

[B12] Doyle M. R. Davis S. J. Bastow R. M. Mcwatters H. G. Kozma-Bognar L. Nagy F. (2002). The ELF4 gene controls circadian rhythms and flowering time in Arabidopsis thaliana. *Nature* 419 74–77. 10.1038/nature00954 12214234

[B13] Faure S. Turner A. S. Gruszka D. Christodoulou V. Davis S. J. Von Korff M. (2012). Mutation at the circadian clock gene EARLY MATURITY 8 adapts domesticated barley (Hordeum vulgare) to short growing seasons. *Proc. Natl. Acad. Sci. U.S.A.* 109 8328–8333. 10.1073/pnas.1120496109 22566625 PMC3361427

[B14] Fujiwara S. Oda A. Yoshida R. Niinuma K. Miyata K. Tomozoe Y. (2008). Circadian Clock Proteins LHY and CCA1 Regulate SVP Protein Accumulation to Control Flowering in Arabidopsis. *Plant Cell* 20 2960–2971. 10.1105/tpc.108.061531 19011118 PMC2613671

[B15] Gendron J. M. Pruneda-Paz J. L. Doherty C. J. Gross A. M. Kang S. E. Kay S. A. (2012). Arabidopsis circadian clock protein, TOC1, is a DNA-binding transcription factor. *Proc. Natl. Acad. Sci. U.S.A.* 109 3167–3172. 10.1073/pnas.1200355109 22315425 PMC3286946

[B16] Gu Z. Huang C. Li F. Zhou X. (2014). A versatile system for functional analysis of genes and microRNAs in cotton. *Plant Biotechnol. J.* 12 638–649. 10.1111/pbi.12169 24521483

[B17] Guo D. Li C. Dong R. Li X. Xiao X. Huang X. (2015). Molecular cloning and functional analysis of the FLOWERING LOCUS T (FT) homolog GhFT1 from Gossypium hirsutum. *J. Integr. Plant Biol.* 57 522–533. 10.1111/jipb.12316 25429737

[B18] Hazen S. P. Schultz T. F. Pruneda-Paz J. L. Borevitz J. O. Ecker J. R. Kay S. A. (2005). LUX ARRHYTHMO encodes a Myb domain protein essential for circadian rhythms. *Proc. Natl. Acad. Sci. U.S.A.* 102 10387–10392. 10.1073/pnas.0503029102 16006522 PMC1177380

[B19] Hsu P. Y. Harmer S. L. (2014). Wheels within wheels: the plant circadian system. *Trends Plant Sci.* 19 240–249. 10.1016/j.tplants.2013.11.007 24373845 PMC3976767

[B20] Hu B. Jin J. Guo A.-Y. Zhang H. Luo J. Gao G. (2015). GSDS 2.0: an upgraded gene feature visualization server. *Bioinformatics* 31 1296–1297. 10.1093/bioinformatics/btu817 25504850 PMC4393523

[B21] Huang H. Nusinow D. A. (2016). Into the Evening: Complex Interactions in the Arabidopsis Circadian Clock. *Trends Genet.* 32 674–686. 10.1016/j.tig.2016.08.002 27594171

[B22] Huang W. Perez-Garcia P. Pokhilko A. Millar A. J. Antoshechkin I. Riechmann J. L. (2012). Mapping the Core of the Arabidopsis Circadian Clock Defines the Network Structure of the Oscillator. *Science* 336 75–79. 10.1126/science.1219075 22403178

[B23] Izawa T. Oikawa T. Sugiyama N. Tanisaka T. Yano M. Shimamoto K. (2002). Phytochrome mediates the external light signal to repress FT orthologs in photoperiodic flowering of rice. *Genes Devel.* 16 2006–2020. 10.1101/gad.999202 12154129 PMC186415

[B24] Kinmonth-Schultz H. A. Golembeski G. S. Imaizumi T. (2013). Circadian clock-regulated physiological outputs: Dynamic responses in nature. *Sem. Cell Devel. Biol.* 24 407–413. 10.1016/j.semcdb.2013.02.006 23435352 PMC3742325

[B25] Li C. Zhang Y. Zhang K. Guo D. Cui B. Wang X. (2015). Promoting flowering, lateral shoot outgrowth, leaf development, and flower abscission in tobacco plants overexpressing cotton FLOWERING LOCUS T (FT)-like gene GhFT1. *Front. Plant Sci.* 6:454. 10.3389/fpls.2015.00454 26136765 PMC4469826

[B26] Li J. Fan S. L. Song M. Z. Pang C. Y. Wei H. L. Li W. (2013). Cloning and characterization of a FLO/LFY ortholog in Gossypium hirsutum L. *Plant Cell Rep.* 32 1675–1686. 10.1007/s00299-013-1479-1 23893068

[B27] Liu C. Qu X. Zhou Y. Song G. Abiri N. Xiao Y. (2018). OsPRR37 confers an expanded regulation of the diurnal rhythms of the transcriptome and photoperiodic flowering pathways in rice. *Plant Cell Environ.* 41 630–645. 10.1111/pce.13135 29314052

[B28] Liu T. L. Newton L. Liu M. J. Shiu S. H. Farre E. M. (2016). A G-Box-Like Motif Is Necessary for Transcriptional Regulation by Circadian Pseudo-Response Regulators in Arabidopsis. *Plant Physiol.* 170 1168–1168. 10.1104/pp.16.000890 26586835 PMC4704597

[B29] Liu X. L. Covington M. F. Fankhauser C. Chory J. Wanger D. R. (2001). ELF3 encodes a circadian clock-regulated nuclear protein that functions in an Arabidopsis PHYB signal transduction pathway. *Plant Cell* 13 1293–1304. 10.1105/tpc.13.6.1293 11402161 PMC135570

[B30] Livak K. J. Schmittgen T. D. (2001). Analysis of relative gene expression data using real-time quantitative PCR and the 2(T)(-Delta Delta C) method. *Methods* 25 402–408. 10.1006/meth.2001.1262 11846609

[B31] Lu S. J. Dong L. D. Fang C. Liu S. L. Kong L. P. Cheng Q. (2020). Stepwise selection on homeologous PRR genes controlling flowering and maturity during soybean domestication. *Nat. Genet.* 52:428. 10.1038/s41588-020-0604-7 32231277

[B32] Lu S. J. Zhao X. H. Hu Y. L. Liu S. L. Nan H. Y. Li X. M. (2017). Natural variation at the soybean J locus improves adaptation to the tropics and enhances yield. *Nat. Genet.* 49:773. 10.1038/ng.3819 28319089

[B33] Lu S. X. Knowles S. M. Andronis C. Ong M. S. Tobin E. M. (2009). CIRCADIAN CLOCK ASSOCIATED1 and LATE ELONGATED HYPOCOTYL Function Synergistically in the Circadian Clock of Arabidopsis. *Plant Physiol.* 150 834–843. 10.1104/pp.108.133272 19218364 PMC2689956

[B34] Lu S. X. Webb C. J. Knowles S. M. Kim S. H. J. Wang Z. Y. Tobin E. M. (2012). CCA1 and ELF3 Interact in the Control of Hypocotyl Length and Flowering Time in Arabidopsis. *Plant Physiol.* 158 1079–1088. 10.1104/pp.111.189670 22190341 PMC3271744

[B35] Madeira F. Park Y. M. Lee J. Buso N. Gur T. Madhusoodanan N. (2019). The EMBL-EBI search and sequence analysis tools APIs in 2019. *Nucl. Acids Res.* 47 W636–W641. 10.1093/nar/gkz268 30976793 PMC6602479

[B36] Marcolino-Gomes J. Nakayama T. J. Molinari H. B. C. Basso M. F. Henning L. M. M. (2017). Functional Characterization of a Putative Glycine max ELF4 in Transgenic Arabidopsis and Its Role during Flowering Control. *Front. Plant Sci.* 8:618. 10.3389/fpls.2017.00618 28473844 PMC5397463

[B37] Marin-Gonzalez E. Matias-Hernandez L. Aguilar-Jaramillo A. E. Lee J. H. Ahn J. H. Suarez-Lopez P. (2015). SHORT VEGETATIVE PHASE Up-Regulates TEMPRANILLO2 Floral Repressor at Low Ambient Temperatures. *Plant Physiol.* 169 1214–1224. 10.1104/pp.15.00570 26243615 PMC4587448

[B38] Matsushika A. Imamura A. Yamashino T. Mizuno T. (2002). Aberrant expression of the light-inducible and circadian-regulated APRR9 gene belonging to the circadian-associated APRR1/TOC1 quintet results in the phenotype of early flowering in Arabidopsis thaliana. *Plant Cell Physiol.* 43 833–843. 10.1093/pcp/pcf118 12198185

[B39] McClung C. R. (2013). Beyond Arabidopsis: The circadian clock in non-model plant species. *Semin. Cell Devel. Biol.* 24 430–436. 10.1016/j.semcdb.2013.02.007 23466287

[B40] McWatters H. G. Bastow R. M. Hall A. Millar A. J. (2000). The ELF3 zeitnehmer regulates light signalling to the circadian clock. *Nature* 408 716–720. 10.1038/35047079 11130072

[B41] McWatters H. G. Kolmos E. Hall A. Doyle M. R. Amasino R. M. Gyula P. (2007). ELF4 is required for oscillatory properties of the circadian clock. *Plant Physiol.* 144 391–401. 10.1104/pp.107.096206 17384164 PMC1913775

[B42] Mizoguchi T. Wheatley K. Hanzawa Y. Wright L. Mizoguchi M. Song H. R. (2002). LHY and CCA1 are partially redundant genes required to maintain circadian rhythms in Arabidopsis. *Devel. Cell* 2 629–641. 10.1016/s1534-5807(02)00170-3 12015970

[B43] Murakami M. Tago Y. Yamashino T. Mizuno T. (2007). Comparative overviews of clock-associated genes of Arabidopsis thaliana and Oryza sativa. *Plant Cell Physiol.* 48 110–121. 10.1093/pcp/pcl043 17132630

[B44] Nagel D. H. Kay S. A. (2012). Complexity in the Wiring and Regulation of Plant Circadian Networks. *Curr. Biol.* 22 R648–R657. 10.1016/j.cub.2012.07.025 22917516 PMC3427731

[B45] Nakamichi N. Kiba T. Henriques R. Mizuno T. Chua N. H. Sakakibara H. (2010). PSEUDO-RESPONSE REGULATORS 9, 7, and 5 Are Transcriptional Repressors in the Arabidopsis Circadian Clock. *Plant Cell* 22 594–605. 10.1105/tpc.109.072892 20233950 PMC2861452

[B46] Nakamichi N. Kiba T. Kamioka M. Suzuki T. Yamashino T. Higashiyama T. (2012). Transcriptional repressor PRR5 directly regulates clock-output pathways. *Proc. Natl. Acad. Sci. U.S.A.* 109 17123–17128. 10.1073/pnas.1205156109 23027938 PMC3479524

[B47] Nakamichi N. Kita M. Ito S. Yamashino T. Mizuno T. (2005). PSEUDO-RESPONSE REGULATORS, PRR9, PRR7 and PRR5, together play essential roles close to the circadian clock of Arabidopsis thaliana. *Plant Cell Physiol.* 46 686–698. 10.1093/pcp/pci086 15767265

[B48] Nakamichi N. Kita M. Niinuma K. Ito S. Yamashino T. Mizoguchi T. (2007). Arabidopsis clock-associated pseudo-response regulators PRR9, PRR7 and PRR5 coordinately and positively regulate flowering time through the canonical CONSTANS-dependent photoperiodic pathway. *Plant Cell Physiol.* 48 822–832. 10.1093/pcp/pcm056 17504813

[B49] Osnato M. Castillejo C. Matias-Hernandez L. Pelaz S. (2012). TEMPRANILLO genes link photoperiod and gibberellin pathways to control flowering in Arabidopsis. *Nat. Commun.* 3:810. 10.1038/ncomms1810 22549837

[B50] Porebski S. Bailey L. G. Baum B. R. (1997). Modification of a CTAB DNA extraction protocol for plants containing high polysaccharide and polyphenol components. *Plant Mole. Biol. Rep.* 15 8–15. 10.1007/bf02772108

[B51] Ronquist F. Huelsenbeck J. P. (2003). MrBayes 3: Bayesian phylogenetic inference under mixed models. *Bioinformatics* 19 1572–1574. 10.1093/bioinformatics/btg180 12912839

[B52] Roy A. Kucukural A. Zhang Y. (2010). I-TASSER: a unified platform for automated protein structure and function prediction. *Nat. Protoc.* 5 725–738. 10.1038/nprot.2010.5 20360767 PMC2849174

[B53] Sato E. Nakamichi N. Yamashino T. Mizuno T. (2002). Aberrant expression of the Arabidopsis circadian-regulated APRR5 gene belonging to the APRR1/TOC1 quintet results in early flowering and hypersensitiveness to light in early photomorphogenesis. *Plant Cell Physiol.* 43 1374–1385. 10.1093/pcp/pcf166 12461138

[B54] Sawa M. Kay S. A. (2011). GIGANTEA directly activates Flowering Locus T in Arabidopsis thaliana. *Proc. Natl. Acad. Sci. U.S.A.* 108 11698–11703. 10.1073/pnas.1106771108 21709243 PMC3136272

[B55] Shim J. S. Kubota A. Imaizumi T. (2017). Circadian Clock and Photoperiodic Flowering in Arabidopsis: CONSTANS Is a Hub for Signal Integration. *Plant Physiol.* 173 5–15. 10.1104/pp.16.01327 27688622 PMC5210731

[B56] Song Y. H. Ito S. Imaizumi T. (2013). Flowering time regulation: photoperiod- and temperature-sensing in leaves. *Trends Plant Sci.* 18 575–583. 10.1016/j.tplants.2013.05.003 23790253 PMC3796012

[B57] Turner A. Beales J. Faure S. Dunford R. P. Laurie D. A. (2005). The pseudo-response regulator Ppd-H1 provides adaptation to photoperiod in barley. *Science* 310 1031–1034. 10.1126/science.1117619 16284181

[B58] Wang L. Sun S. Wu T. Liu L. Sun X. Cai Y. (2020). Natural variation and CRISPR/Cas9-mediated mutation inGmPRR37affect photoperiodic flowering and contribute to regional adaptation of soybean. *Plant Biotechnol. J.* 18 1869–1881. 10.1111/pbi.13346 31981443 PMC7415786

[B59] Wang Z. Y. Tobin E. M. (1998). Constitutive expression of the CIRCADIAN CLOCK ASSOCIATED 1 (CCA1) gene disrupts circadian rhythms and suppresses its own expression. *Cell* 93 1207–1217. 9657153 10.1016/s0092-8674(00)81464-6

[B60] Wohlbach D. J. Thompson D. A. Gasch A. P. Regev A. (2009). From elements to modules: regulatory evolution in Ascomycota fungi. *Curr. Opin. Genet. Devel.* 19 571–578. 10.1016/j.gde.2009.09.007 19879128 PMC2853222

[B61] Yakir E. Hilman D. Kron I. Hassidim M. Melamed-Book N. Green R. M. (2009). Posttranslational Regulation of CIRCADIAN CLOCK ASSOCIATED1 in the Circadian Oscillator of Arabidopsis. *Plant Physiol.* 150 844–857. 10.1104/pp.109.137414 19339503 PMC2689986

[B62] Yamamoto Y. Sato E. Shimizu T. Nakamich N. Sato S. Kato T. (2003). Comparative genetic studies on the APRR5 and APRR7 genes belonging to the APRR1/TOC1 quintet implicated in circadian rhythm, control of flowering time, and early photomorphogenesis. *Plant Cell Physiol.* 44 1119–1130. 10.1093/pcp/pcg148 14634148

[B63] Zagotta M. T. Hicks K. A. Jacobs C. I. Young J. C. Hangarter R. P. Meekswagner D. R. (1996). The Arabidopsis ELF3 gene regulates vegetative photomorphogenesis and the photoperiodic induction of flowering. *Plant J.* 10 691–702. 10.1046/j.1365-313X.1996.10040691.x 8893545

[B64] Zhang R. Ding J. Liu C. X. Cai C. P. Zhou B. L. Zhang T. Z. (2015). Molecular Evolution and Phylogenetic Analysis of Eight COL Superfamily Genes in Group I Related to Photoperiodic Regulation of Flowering Time in Wild and Domesticated Cotton (Gossypium) Species. *PLoS One* 10:e0118669. 10.1371/journal.pone.0118669 25710777 PMC4339614

[B65] Zhang T. Hu Y. Jiang W. Fang L. Guan X. Chen J. (2015). Sequencing of allotetraploid cotton (Gossypium hirsutum L. acc. TM-1) provides a resource for fiber improvement. *Nat. Biotechnol.* 33 531–537. 10.1038/nbt.3207 25893781

[B66] Zhao J. Huang X. Ouyang X. Chen W. Du A. Zhu L. (2012). OsELF3-1, an Ortholog of Arabidopsis EARLY FLOWERING 3, Regulates Rice Circadian Rhythm and Photoperiodic Flowering. *PLoS One* 7:e043705. 10.1371/journal.pone.0043705 22912900 PMC3422346

