# Supplementary material for: GhLUX1 and GhELF3 Are Two Components of the Circadian Clock That Regulate Flowering Time of Gossypium hirsutum
Source: Front Plant Sci. 2021 Aug 9;12:691489. doi: 10.3389/fpls.2021.691489 (PMC8380988; doi:10.3389/fpls.2021.691489)
Supplement: Supplementary Figure 1 — Exon-intron structures of GhLUX1 and GhELF3. [file Data_Sheet_1.PDF]

Supplementary Material

1 Supplementary Figures

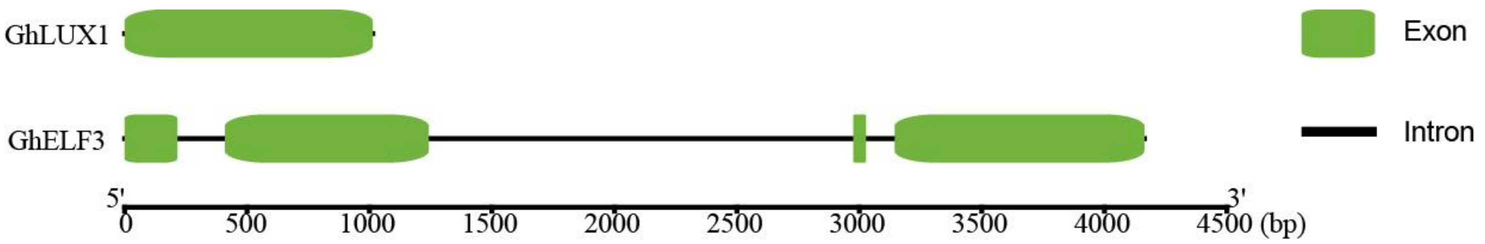

Figure S1. Exon-intron structures of GhLUX1 and GhELF3. Exons and introns are indicated by the rounded rectangles and lines, respectively.

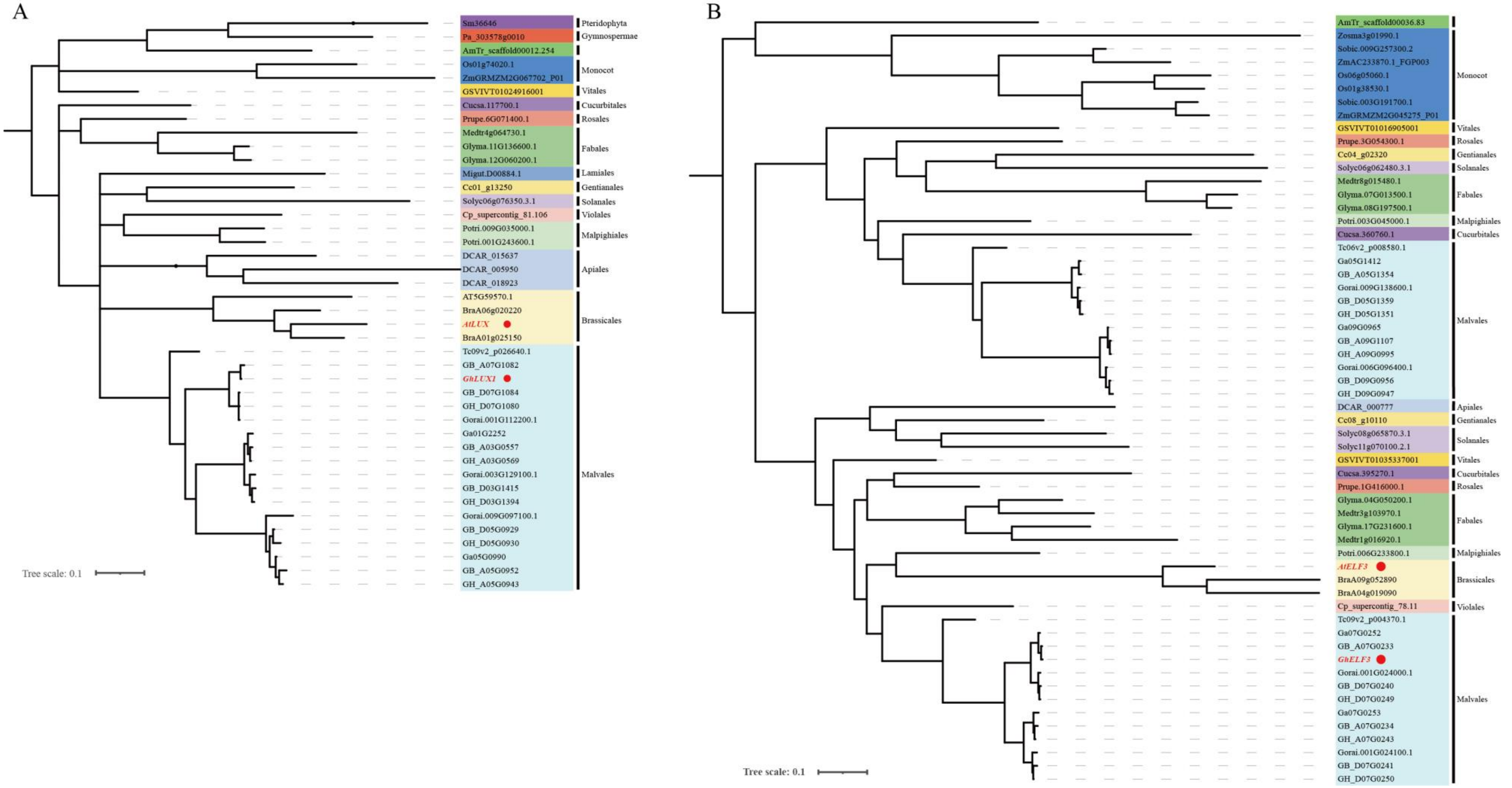

Figure S2. Phylogenetic trees of LUXs and ELF3s in plant species. (A) Phylogenetic tree of LUXs. (B) Phylogenetic tree of ELF3s. The phylogenetic trees of identified LUXs and ELF3s are constructed by using MrBayes v3.2.5. LUXs and ELF3s in different Classes or Orders are highlighted with different background colors and the names of Classes or Orders are indicated. LUXs and ELF3s in Arabidopsis and G. hirsutum are colored red and marked with filled circles. The tree scale bar represents 0.1 substitutions per amino acid.

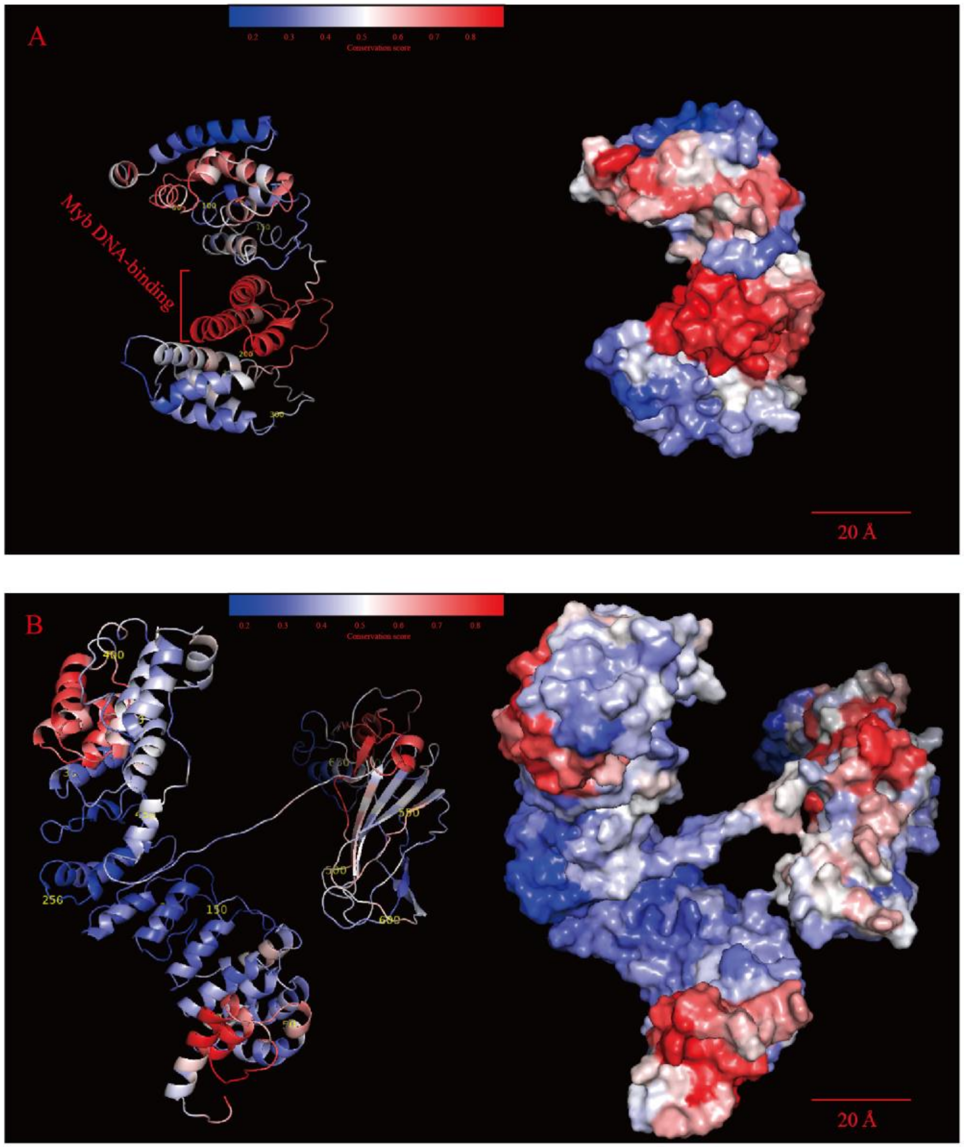

Figure S3. Tertiary structures of GhLUX1 and GhELF3. (A) Tertiary structure of GhLUX1. (B) Tertiary structure of GhELF3. The left and right side are the proteins' interior and surface structures, respectively. The conservation score at each amino acid site is mapped to the gradient from blue to red with blue representing low conservation score and red representing high conservation score. The Myb DNA-binding domain of GhLUX1 is indicated with red text at the corresponding location.

2 Supplementary Tables

Table S1. Properties of the putative protein sequences of GhLUX1 and GhELF3

Name	Length (aa)	MW (Da)	pI	GRAVY
GhLUX1	337	36451.16	5.28	-0.715
GhELF3	706	76360.41	8.84	-0.726

Table S2. Primers used in this study

Name	Use	Sequence (5' to 3')
GhLUX1-OE-F	Overexpression	cacgggggactctagaATGGGTCAAGAAGTGAAGATG
GhLUX1-OE-R	Overexpression	gatcggggaaattcgagctcTCATTGGGAACCATATCTATT
GhELF3-OE-F	Overexpression	cacgggggactctagaATGAAGAGAGGAAAAGATGAT
GhELF3-OE-R	Overexpression	gatcggggaaattcgagctcCTCATCTTAGTCCCGTTGCTC
GhLUX1-qPCR-F	qPCR	GAAGCGGAAGGATATGGGTCGG
GhLUX1-qPCR-R	qPCR	CGGTCGTTTCAATGTCCTCCCA
GhELF3-qPCR-F	qPCR	TGTGCCAAAGAGGCCAATGTGT
GhELF3-qPCR-R	qPCR	CCACAAGTCCTGTCTTCATGGCA
GhFT-qPCR-F	qPCR	AGAGATCCTTTGGTTGTTGGT
GhFT-qPCR-R	qPCR	CCTCAGATCATCCCCACCAAT
GhCOL1-qPCR-F	qPCR	CCTGCTGCCCTGTTGTGCAAG
GhCOL1-qPCR-R	qPCR	ATTGGAAGAATGGGGACACGC
GhActin-qPCR-F	qPCR	ATCCTCCGTCTTGACCTTG
GhActin-qPCR-R	qPCR	TGTCCGTCAGGCAACTCAT
AtLUX-qPCR-F	qPCR	GATGCCAGTTTATGCACATCAT
AtLUX-qPCR-R	qPCR	GAACAGATGCAATTTGGGACTT
AtELF3-qPCR-F	qPCR	GCTTCATGTGAATGATGCAGAT
AtELF3-qPCR-R	qPCR	AAGAATCAAGATGCTGGACAGA
AtELF4-qPCR-F	qPCR	GGCTGATAACATGTCGAAGAAC
AtELF4-qPCR-R	qPCR	CCCGACGAGAAACTAGTATTGA

AtPRR7-qPCR-F	qPCR	CCAAGTGGTACATTCTAACCCT
AtPRR7-qPCR-R	qPCR	GCTAATCTCCAAGTCTCTACCC
AtLHY-qPCR-F	qPCR	CAGGATGATTACCGTTCGTTTC
AtLHY-qPCR-R	qPCR	GCAATGGCAGTTATACTTGGAG
AtCCA1-qPCR-F	qPCR	TTTACAACATGGTTCTGTGCAG
AtCCA1-qPCR-R	qPCR	GGTCAACTTGTTTTCTGTCTCC
AtFT-qPCR-F	qPCR	CTACAACTGGAACAACCTTTGG
AtFT-qPCR-R	qPCR	TGACAATTGTAGAAAACTGCGG
AtCO-qPCR-F	qPCR	GGAGATAGAGTTGTTCCGCTTA
AtCO-qPCR-R	qPCR	CCATGGATGAAATGTATGCGTT
AtTEM1-qPCR-F	qPCR	CGATGAGTTTGAGCAGAGTAGA
AtTEM1-qPCR-R	qPCR	TTCGGTATCACTAAACGGTTCA
AtGI-qPCR-F	qPCR	CTGTTTAAACTGGGAAGCTCAC
AtGI-qPCR-R	qPCR	GGGACAAGGATATAGTACAGCC
AtFKF1-qPCR-F	qPCR	GATGATGGAACCATTACACACG
AtFKF1-qPCR-R	qPCR	TTTGAACACAGGATACGAGACA
AtACT2-qPCR-F	qPCR	CTCCTTTGTTGCTGTTGACTAC
AtACT2-qPCR-R	qPCR	GCACAATGTTACCGTACAGATC
GhLUX1-BD-F	Y2H	catggaggccgaattcATGGGTCAAGAAGTGAAGATG
GhLUX1-BD-R	Y2H	gcaggtcgacggatccTCATTGGGAACCATATCTATT
GhELF3-BD-F	Y2H	catggaggccgaattcATGAAGAGAGGAAAAGATGAT
GhELF3-BD-R	Y2H	gcaggtcgacggatccTAGTCCCGTTGCTCTCTCTTT
GhLUX1N-BD-F	Y2H	catggaggccgaattcATGGGTCAAGAAGTGAAGATG
GhLUX1N-BD-R	Y2H	gcaggtcgacggatccGTTCTCCGTCGTCCTAACCG
GhLUX1C-BD-F	Y2H	catggaggccgaattcTCGGACGATCCGTCTGGGAG
GhLUX1C-BD-R	Y2H	gcaggtcgacggatccTCATTGGGAACCATATCTATT
GhELF3-AD-F	Y2H	ggaggccagtgaattcATGAAGAGAGGAAAAGATGAT
GhELF3-AD-R	Y2H	cgagctcgatggatccTAGTCCCGTTGCTCTCTCTTT
GhELF3N-AD-F	Y2H	ggaggccagtgaattcATGAAGAGAGGAAAAGATGAT
GhELF3N-AD-R	Y2H	cgagctcgatggatccTGGTGAAGGTGGTTTATTTCC
GhELF3C-AD-F	Y2H	ggaggccagtgaattcATGAACCCTTGGTGTTTCAAT

GhELF3C-AD-R	Y2H	cgagctcgatggatccTAGTCCCGTTGCTCTCTCTTT
GhLUX1-GFP-F	Subcellular localization	cacgggggactctagaATGGGTCAAGAAGTGAAGATG
GhLUX1-GFP-R	Subcellular localization	ctttactcatactagtGGAACCATATCTATTCCCATC
GhELF3-GFP-F	Subcellular localization	cacgggggactctagaATGAAGAGAGGAAAAGATGAT
GhELF3-GFP-R	Subcellular localization	ctttactcatactagtTAGTCCCGTTGCTCTCTCTTT
GhELF3-NE-F	BiFC	gcctactagtggatccATGAAGAGAGGAAAAGATGAT
GhELF3-NE-R	BiFC	gagcggtaccctcgagTCATCTTAGTCCCGTTGCTC
GhLUX1-CE-F	BiFC	cgccactagtggatccATGGGTCAAGAAGTGAAGATG
GhLUX1-CE-R	BiFC	gagcggtaccctcgagGGAACCATATCTATTCCCATC
GhLUX1-VIGS-F	VIGS	caaaatggcatgcctgcagactagtGTTGATGTCAACCGCGCTTCT
GhLUX1-VIGS-R	VIGS	gaattcactagacctaggggcgcgccCAACGAATCTCTTGTGCAGCT
GhELF3-VIGS-F	VIGS	caaaatggcatgcctgcagactagtCCTGATGAAACTAGTAATGAC
GhELF3-VIGS-R	VIGS	gaattcactagacctaggggcgcgccAGTATCCTCAAGCAACAGATG

Table S3. Information about the 27 plant species used in identifying LUXs and ELF3s

**Scientific name**	**Abbreviation**	**URLs**	**Genome version**
Amborella trichopoda	AmTr	https://phytozome.jgi.doe.gov/pz/portal.html	v1.0
Arabidopsis thaliana	At	https://phytozome.jgi.doe.gov/pz/portal.html	TAIR10
Brassica rapa	Bra	http://brassicadb.org/	v3.0
Carica papaya	Cp	https://phytozome.jgi.doe.gov/pz/portal.html	ASGPBv0.4
Coffea canefora	Cc	http://coffee-genome.org/	v1.0
Chlamydomonas reinhardtii	Cre	https://phytozome.jgi.doe.gov/pz/portal.html	v5.6
Cucumis sativus	Cucsa	https://phytozome.jgi.doe.gov/pz/portal.html	v1.0
Daucus carota	DCAR	https://phytozome.jgi.doe.gov/pz/portal.html	v2.0
Glycine max	Glyma	https://phytozome.jgi.doe.gov/pz/portal.html	Wm82.a2.v1
Gossypium arboreum	Ga	https://www.cottongen.org/data/download/genome_diploid_A_nd_D	A2_CRI
Gossypium barbadense	GB	https://www.cottongen.org/data/download/genome_tetraploid/AD2	ZJU_H7124
Gossypium hirsutum	GH	https://www.cottongen.org/data/download/genome_tetraploid/AD1	ZJU_v2.1

Gossypium raimondii	Gorai	https://www.cottongen.org/data/download/genome_diploid_A_nd_D	D5_JGI
Medicago truncatula	Medtr	https://phytozome.jgi.doe.gov/pz/portal.html	Mt4.0v1
Mimulus guttatus	Migut	https://phytozome.jgi.doe.gov/pz/portal.html	v2.0
Oryza sativa	Os	https://phytozome.jgi.doe.gov/pz/portal.html	v7.0
Physcomitrella patens	Pp	https://phytozome.jgi.doe.gov/pz/portal.html	v3.3
Picea abies	Pa	http://congenie.org/	v1.0
Populus trichocarpa	Potri	https://phytozome.jgi.doe.gov/pz/portal.html	v3.0
Prunus persica	Prupe	https://phytozome.jgi.doe.gov/pz/portal.html	v2.1
Selaginella moellendorffii	Sm	https://phytozome.jgi.doe.gov/pz/portal.html	v1.0
Solanum lycopersicum	Solyc	https://phytozome.jgi.doe.gov/pz/portal.html	ITAG3.2
Sorghum bicolor	Sobic	https://phytozome.jgi.doe.gov/pz/portal.html	v3.1
Theobroma cacao	Tc	https://cocoa-genome-hub.southgreen.fr/	CriolloB97_v2
Vitis vinifera	GSVIVT	https://phytozome.jgi.doe.gov/pz/portal.html	Genoscope.12X
Zea mays	Zm	https://phytozome.jgi.doe.gov/pz/portal.html	Ensembl-18_2010-01-MaizeSequence
Zostera marina	Zosma	https://phytozome.jgi.doe.gov/pz/portal.html	v2.2
